# Impact of Maternal Education on Perinatal and Maternal Outcomes in Women With a History of Gestational Diabetes

**DOI:** 10.7759/cureus.83044

**Published:** 2025-04-26

**Authors:** Karla Collazo Melian, Bianca Taylor, Yulio Font, Grettel Castro, Marcia Varella

**Affiliations:** 1 College of Medicine, Florida International University, Herbert Wertheim College of Medicine, Miami, USA; 2 Department of Medical and Population Health Sciences Research, Florida International University, Herbert Wertheim College of Medicine, Miami, USA

**Keywords:** congenital anomalies (ca), gestational diabetes, health literacy, maternal icu admission, perinatal complications

## Abstract

Introduction: Gestational diabetes is a common complication of pregnancy and is linked to adverse health outcomes for both mothers and their children. Lower maternal educational levels and poor health literacy have also been associated with poor health outcomes. However, few studies have analyzed the magnitude of the association between maternal education and poor outcomes for both mother and child in the context of gestational diabetes. This study aimed to explore the potential relationship between maternal and paternal education levels and the risk of negative health outcomes in both mother and child in pregnant women with gestational diabetes in the United States.

Methods: This project is a retrospective cohort study. National Vital Statistics System-Natality (NVSS-N) data from 2019 to 2020 was used, sampling women aged 18-34 with gestational diabetes who gave birth to a singleton. Outcomes were the presence of at least one congenital anomaly and maternal intensive care unit (ICU) admission. Multivariable logistic regression analysis via Stata V. 16 (StataCorp LLC, College Station, TX, US) estimated adjusted odds ratio (OR) and 95% confidence interval (CI).

Results: Among 541,117 women, 38% had a high school diploma or less, 52% completed some college, and 11% held a master's degree or higher. The maternal ICU admission rate was 0.17%, and at least one congenital anomaly occurred in 0.21% of births. In adjusted models, compared to mothers with a high school diploma or less, those with a master's degree or some college had adjusted ORs of 1.14 (95% CI = 0.80, 1.61) and 1.11 (95% CI = 0.90, 1.36) for congenital anomalies, respectively. Paternal education was associated with reduced odds for both outcomes.

Conclusions: Maternal education was not associated with adverse outcomes in gestational diabetes. Findings suggest paternal education may impact patient outcomes and should be further explored. Potential alternative targets to improve the health of women with gestational diabetes and their offspring are warranted.

## Introduction

Gestational diabetes mellitus is the most prevalent disease among pregnant women, affecting up to 15%-25% of pregnancies worldwide [1]. Gestational diabetes prevalence has increased among all races, ethnicities, and age groups from 47.6 per 1,000 live births in 2011 to 63.5 in 2019. In the United States, the National Center for Health Statistics estimated an increase in gestational diabetes prevalence of 3.7% per year in that same period [2].

Women who experience gestational diabetes have a 20%-60% increased risk of developing type 2 diabetes within five to 10 years post-delivery. These women also had a 2% increased risk for preeclampsia, cesarean section, and preterm delivery [3]. Gestational diabetes is also shown to be associated with an increased risk of several congenital anomalies in the newborn [4]. Other adverse pregnancy outcomes reported include fetal macrosomia, cesarean sections, stillbirth, and premature induction of labor, and offspring are more likely to develop diabetes and obesity later in life [3]. Considering the detrimental consequences of gestational diabetes, strategies to improve prognosis are key to improving the quality of health of women with that condition and their children. While many clinical factors are targeted with intervention, other less traditional modifiable contributing factors are not as frequently assessed. One of such factors is education. Lower education levels have been associated with reduced health literacy in pregnant women. As a result of reduced health literacy, pregnant women may struggle to thoroughly understand the health complications, the importance of prenatal care, and medication adherence during pregnancy [5]. Consistent with this claim, a multinational, cross-sectional, and internet-based study performed in 18 countries, including the United States, found that low health literacy in women was more often characterized by negative beliefs about medications that should be taken during pregnancy [6]. It has also been found that a high prevalence of low health literacy impacts patient understanding of the importance of first- and second-trimester fetal screening tests, such as fetal aneuploidy and neural tube defects [7]. In Italy, a cohort study from 2005 to 2010 found that mothers with an intermediate level of education were associated with slightly lower odds of severe congenital abnormalities compared to mothers with lower levels of education. However, a higher education level was not related to the odds of severe congenital abnormalities [8]. In a meta-analysis conducted in 2021, increased parental education reduced by 31% the risk of child mortality globally [9]. Yet, the magnitude of the impact of education in the context of gestational diabetes was not explored in those studies.

In the specific setting of women with gestational diabetes, a cross-sectional study of 104 Iranian pregnant women linked low education levels and reduced health literacy with poor glycemic control during pregnancy [10]. Women with lower health literacy and gestational diabetes presented with an average Hb1Ac of 7.1 as compared to 6.5 in those with adequate health literacy, indicating a poor understanding of methods to regulate hyperglycemic levels. It has also been found that only 31% of pregnant women with pregestational diabetes and low health literacy took the necessary prenatal vitamins, like folic acid, as compared to 83% of women with adequate health literacy [11]. Ultimately, they found that 69% of women found to have lower health literacy were hospitalized during pregnancy, compared to only 36% of women with adequate levels of health literacy [11].

While evidence suggests that levels of education are associated with maternal and newborn detrimental outcomes, not many studies have been conducted specifically on mothers with gestational diabetes, particularly in the United States. This study aimed to add to the current literature by evaluating the association between lower education and risk of negative health outcomes in both mother and child in pregnant women with gestational diabetes in the United States using the National Vital Statistics System (NVSS) registry from 2019 to 2020. Paternal educational attainment was included as an independent variable to explore its potential impact on perinatal and maternal outcomes.

This study was presented at the David A. Paulus, MD Poster Symposium of the 2024 Florida Medical Association on August 3, 2024.

## Materials and methods

Design and setting

The study is a non-concurrent cohort using data from the US Standard Certificates of Live Birth from the NVSS-Natality (NVSS-N) from January 1, 2019, to December 31, 2020.

Participants

We included women aged 18-34 with gestational diabetes who gave birth from 2019 to 2020 and their newborns as reported by the NVSS. Women with reported pregestational diabetes, plural deliveries, or previous stillbirths were excluded. At the analysis phase, we further excluded women who lacked information concerning maternal education, congenital abnormalities, or maternal stays in the intensive care unit (ICU).

Variables

All variables were drawn directly or indirectly from responses to questions in the US Standard Certificate of Live Birth. The independent variable was the mother's highest level of educational achievement at the time of delivery. Mother's education will be recorded under the following possible values: (1) high school (HS) diploma, (2) some college, and (3) master’s degree. To operationally define these variables, HS diploma included those with no formal HS education, some HS, or a completed HS diploma or General Educational Development (GED). Some college included individuals with a partial college education, an associate degree, or a bachelor’s degree. The master’s degree category included individuals who completed graduate-level education (master’s or doctoral degrees).

The two main dependent variables were (1) maternal admission to the ICU and (2) the presence of any the following congenital anomalies of the newborn: anencephaly, meningomyelocele/spina bifida, cyanotic congenital heart disease, congenital diaphragmatic hernia, omphalocele, gastroschisis, limb reduction defect (excluding congenital amputation and dwarfing syndrome), cleft lip with or without cleft palate, and cleft palate alone as recorded in the database. Down syndrome is not included as a congenital malformation for this outcome. Maternal and newborn outcomes were assessed separately. Confounders assessed included the mother's age at delivery, race, state of residence, body mass index (BMI), trimester prenatal care began, and delivery payment source (Medicaid, private insurance, self-pay, or other). We assessed effect modification by paternal education levels categorized as HS, some college, and a master's degree.

Statistical analysis plan

All statistical analyses were conducted using Stata V. 16 (StataCorp LLC, College Station, TX, US). We performed a descriptive univariate analysis to assess the distribution and completeness of all variables, followed by bivariate analyses to evaluate the association between all potential confounders with both the exposure and, subsequently, each of the outcomes. Significance was assessed using a chi-squared test for categorical variables. Finally, binary logistic regression was used to calculate unadjusted and adjusted odds ratios (ORs) and corresponding 95% confidence intervals (CIs). The regression was performed separately for each of the outcomes assessed. A test of effect modification was performed using interaction terms in the regression models. A p-value of less than 0.05 was deemed statistically significant for all analyses.

Ethical aspects

The ethical implications of this research project are minimal. The study utilized data from the NVSS-N, a publicly available and fully de-identified database. Because the dataset is anonymized and the research is retrospective in nature, Institutional Review Board (IRB) approval and informed consent were not required. All data used for analysis were free of patient identifiers, and no protected health information was accessed.

## Results

The number of women aged 18-34 with gestational diabetes who gave birth from January 1, 2019, to December 31, 2020, as reported by the NVSS-N was 541,117.

Distribution of maternal characteristics by educational attainment

Table 1 shows the distribution of characteristics according to maternal educational attainment. Selected characteristics were associated with maternal educational attainment. For instance, as the level of maternal educational attainment increases, women were more likely to be older and of a non-Hispanic White race and US-born, be more likely to have lower BMI, have a partner with a higher level of educational attainment, start prenatal care in the first trimester, and pay with private insurance and less likely to use the Women, Infants, and Children Program (WIC). In contrast, women who attained a lower educational level were more likely to be of Hispanic race, be obese, have a partner with lower educational attainment, and utilize WIC services and Medicaid, as compared to women of higher educational attainment.

**Table 1:** Characteristics of women with gestational diabetes who gave birth from 2019 to 2020 by the highest level of education achieved p-values for the chi-squared test for comparisons across educational levels N: total number of women; %: percentage out of each educational category; BMI: body mass index; WIC: Women, Infants, and Children Program

	Maternal education						
Characteristics	High school		Some college		Master's		p-value
	N	%	N	%	N	%	
Maternal age							<0.001
18-19	5,348	3.9	768	0.4	0	0	
20-24	33,179	24.6	20,498	11.5	215	0.6	
25-29	47,825	35.4	65,993	37.1	7,865	22.2	
30-34	48,581	36.0	90,528	50.9	27,377	77.2	
Maternal race							<0.001
Non-Hispanic White	51,173	38.1	96,046	54.2	19,504	55.2	
Non-Hispanic Black	18,644	13.9	20,280	11.4	2,166	6.1	
Hispanic	51,181	38.1	35,123	19.8	2,724	7.7	
Other	13,503	10.0	25,788	14.6	10,960	31.0	
Maternal nationality							<0.001
US-born	93,223	69.2	140,718	79.3	22,966	64.8	
Non-US-born	41,427	30.8	36,825	20.7	12,456	35.2	
Maternal BMI							<0.001
Underweight	2,056	1.6	2,475	1.4	756	2.2	
Normal	25,706	19.4	42,235	24.1	13,888	39.7	
Overweight	32,718	24.7	44,802	25.5	9,673	27.7	
Obese	71,869	54.3	85,888	48.9	10,632	30.4	
Father's education							<0.001
High school	88,857	80.00	50,869	31.0	2,869	8.2	
Some college	21,276	19.2	99,863	60.9	17,953	51.4	
Master's	942	0.9	13,200	8.1	14,119	40.4	
Maternal WIC status							<0.001
Yes	75,112	56.2	47,053	26.7	1,904	5.4	
No	58,603	43.8	129,224	73.3	33,198	94.6	
Maternal pay (insurance)							<0.001
Medicaid	91,805	68.4	57,455	32.5	2,409	6.8	
Private insurance	32,504	24.2	109,238	61.8	31,476	89.2	
Self-pay	5,514	4.1	3,086	1.8	547	1.6	
Other	4,402	3.3	6,944	3.9	868	2.5	
Prenatal care initiation (month)							<0.001
1-3	97,775	73.7	147,118	83.94	31,242	89.3	
4-6	26,894	20.3	21,767	12.4	2,777	7.9	
7-9	6,752	5.1	5,589	3.2	849	2.4	
None	1,198	0.9	793	0.5	115	0.3	

The data in Table 1 are presented as those who were self-reported in the NVSS and are intended for descriptive purposes only.

Relationship between maternal educational attainment and the occurrence of congenital anomalies and maternal ICU admissions

Table 2 shows the frequency of congenital anomalies according to maternal covariates. The frequency of at least one congenital anomaly occurrence decreased as the level of maternal educational attainment increased, yet this difference was not statistically significant (p = 0.153). Also, as maternal age increases, the frequency of birthing a child with at least one congenital anomaly decreases. Non-Hispanic Whites birthed children at the highest frequencies of congenital anomalies, and "other race" birthed children at the lowest (0.25% and 0.18%, respectively, p = 0.002). US-born mothers birthed children with at least one congenital anomaly at a higher frequency when compared to non-US-born mothers (0.23% vs. 0.17%, respectively, p = 0.001). Women partnered with fathers with higher educational attainment birthed children with at least one congenital anomaly at a lower frequency (0.23% vs. 0.20% vs. 0.16%, HS or less vs. bachelor's degree or less vs. master's degree or more, respectively, p = 0.032). Women who began prenatal care earlier birthed children with at least one congenital anomaly at lower frequencies than those who started prenatal care later. However, women who received no prenatal care birthed children with at least one congenital anomaly at a lower frequency than those who began prenatal care in the third trimester (0.18% vs. 0.30% vs. 0.44% vs. 0.42%, first trimester vs. second trimester vs. third trimester vs. none, respectively).

**Table 2:** Frequency of congenital anomalies in newborns of women with gestational diabetes and maternal ICU admission in those who gave birth from 2019 to 2020 p-values for the chi-squared test for comparisons of frequencies across subcategories of the selected characteristics N: total number of women; BMI: body mass index; WIC: Women, Infants, and Children Program; ICU: intensive care unit

Characteristics	Congenital anomaly			p-value	Maternal ICU admission			p-value
	None	N	N/100,000		None	N	N/100,000	
Maternal education				0.153				0.022
High school	134,625	308	23		134,658	285	21	
Some college	177,406	381	21		177,486	301	17	
Master's	35,395	62	17		35,396	61	17	
Maternal age				0.031				0.174
18-19	6,130	19	31		6,133	16	26	
20-24	54,008	139	26		54,056	91	17	
25-29	122,067	262	21		122,115	214	17	
30-34	167,366	334	20		167367	333	20	
Maternal race				0.002				0.073
Non-Hispanic White	166,677	411	25		166,795	293	18	
Non-Hispanic Black	41,132	76	18		41,110	98	24	
Hispanic	89,349	178	20		89,362	165	18	
Other	50,419				50,411	95	19	
Maternal nationality				0.001				0.434
US-born	257,398	597	23		257,506	489	19	
Non-US-born	91,587	157	17		91,582	162	18	
Maternal BMI				0.284				0.033
Underweight	5,315	12	23		5,316	11	21	
Normal	82,201	168	20		82,250	119	14	
Overweight	87,544	168	19		87,555	167	19	
Obese	168,877	384	23		168,929	332	20	
Father's education				0.032				0.002
High school	142,467	323	23		142,513	277	19	
Some college	138,949	274	20		139,006	217	16	
Master's	28,242	44	16		28,355	31	11	
Maternal WIC status				0.345				0.08
Yes	124,578	281	23		124,545	254	20	
No	221,783	466	21		221,856	393	18	
Maternal pay (insurance)				0.202				0.031
Medicaid	152,320	351	23		152,360	311	17	
Private insurance	173,812	345	20		173,863	294	23	
Self-pay	9,209	22	24		9,206	25	20	
Other	12,275	30	24		12,283	22	75	
Prenatal care initiation (month)				<0.001				<0.001
1-3	277,308	506	18		277,348	466	20	
4-6	51,574	153	30		51,610	117	17	
7-9	13,232	59	44		13,265	26	27	
None	2,119	9	42		2,122	16	18	

Additionally, Table 2 displays the frequency of maternal ICU admissions according to selected characteristics. The frequency of maternal ICU admission decreased with higher levels of maternal education (0.21% vs. 0.17% vs. 0.17% for HS or less vs. bachelor's degree or less vs. master's degree or more, respectively, p = 0.022). Likewise, the frequency of maternal ICU admission decreased with higher paternal educational attainment (0.19% vs. 0.16% vs. 0.11% for HS or less vs. bachelor's degree or less vs. master's degree or more, respectively, p = 0.002). Both extremes of maternal BMI had increased frequency of maternal ICU admissions, with underweight mothers having the highest rates of ICU admission (0.21%, p = 0.033), followed by obese women (0.20%, p = 0.033). Additionally, late-term or lack of prenatal care had increased rates of maternal ICU admissions. Women who received no prenatal care had the highest rates of maternal ICU admission (0.18%, p = 0.001), followed by women who received prenatal care in the second trimester (0.17%, p = 0.001) and third trimester (0.27%, p = 0.001). Mothers who used self-pay as a form of payment also had the highest rates of ICU admission (0.27%, p = 0.031), followed by mothers who used Medicaid (0.20%, p = 0.031). However, statistically significant results were not found between maternal ICU admission and maternal age, race, nationality, or WIC status.

Associations between maternal education and other characteristics with the development of congenital anomalies

Table 3 displays the associations between congenital anomalies and selected characteristics. We did not find a statistically significant relationship between maternal educational attainment and birthing a child with at least one congenital anomaly in either the adjusted or unadjusted models. Results of the adjusted analyses suggested significant associations between maternal race and congenital anomalies. Non-Hispanic Black mothers had the lowest odds of birthing a child with at least one congenital anomaly (OR = 0.62, 95% CI = 0.45, 0.85), followed by other (OR = 0.74, 95% CI = 0.55, 0.99), and finally, Hispanic (OR = 0.80, 95% CI = 0.64, 0.99), when compared to non-Hispanic White mothers. Non-US-born mothers had lower adjusted odds of congenital anomalies when compared to US-born mothers (OR = 0.77, 95% CI = 0.61, 0.97). Children of fathers with bachelor's degrees and less (OR = 0.79, 95% CI = 0.63, 0.98) and master's degrees or more (OR = 0.52, 95% CI = 0.33, 0.80) displayed lower odds of congenital anomalies than their counterparts of fathers who completed HS or less. Finally, women who began their prenatal care in the second trimester displayed higher adjusted odds of congenital anomalies than mothers who began prenatal care in the first trimester (OR = 1.30, 95% CI = 1.02, 1.65).

**Table 3:** Unadjusted and adjusted associations between maternal education, selected characteristics, and occurrence of any congenital anomalies Unadjusted and adjusted analyses of congenital anomalies; binary logistic regression for calculation of unadjusted and adjusted ORs and corresponding 95% CIs BMI: body mass index; WIC: Women, Infants, and Children Program; OR: odds ratio; CI: confidence interval

Characteristics	Unadjusted		Adjusted	
	OR (95% CI)	p-value	OR (95% CI)	p-value
Maternal education				
High school	Reference	Reference	Reference	Reference
Some college	0.9 (0.81, 1.09)	0.410	1.1 (0.90, 1.36)	0.309
Master's	0.8 (0.58, 1.01)	0.055	1.1 (0.80, 1.61)	0.473
Maternal age				
18-19	1.4 (0.91, 2.30)	0.123	0.9 (0.44, 1.68)	0.658
20-24	1.2 (0.98, 1.47)	0.084	0.9 (0.78, 1.26)	0.930
25-29	Reference		Reference	Reference
30-34	0.9 (0.79, 1.09)	0.000	0.9 (0.77, 1.10)	0.357
Maternal race				
Non-Hispanic White	Reference	Reference	Reference	Reference
Non-Hispanic Black	0.6 (0.59, 0.96)	0.021	0.6 (0.45, 0.85)	0.003
Hispanic	0.8 (0.68, 0.96)	0.018	0.8 (0.64, 0.99)	0.041
Other	0.7 (0.55, 0.88)	0.003	0.7 (0.55, 0.99)	0.041
Maternal nationality				
US-born	Reference	Reference	Reference	Reference
Non-US-born	0.7 (0.62, 0.88)	0.001	0.8 (0.61, 0.97)	0.030
Maternal BMI				
Underweight	1.1 (0.61, 1.99)	0.739	1.3 (0.69, 2.35)	0.447
Normal	Reference	Reference	Reference	Reference
Overweight	0.9 (0.76, 1.16)	0.564	0.9 (0.72, 1.16)	0.448
Obese	1.1 (0.93, 1.33)	0.249	1.1 (0.85, 1.29)	0.654
Father's education				
High school	Reference	Reference	Reference	Reference
Some college	0.9 (0.74, 1.02)	0.900	0.8 (0.63, 0.98)	0.034
Master's	0.7 (0.50, 0.94)	0.020	0.5 (0.33, 0.80)	0.003
Maternal WIC status				
Yes	1.1 (0.92, 1.25)	0.345	1.07 (0.86, 1.34)	0.539
No	Reference	Reference	Reference	Reference
Maternal pay (insurance)				
Medicaid	1.2 (1.00, 1.35)	0.049	0.9 (0.72, 1.16)	0.477
Private insurance	Reference	Reference	Reference	Reference
Self-pay	1.2 (0.78, 1.85)	0.400	1.3 (0.77, 2.12)	0.338
Other	1.2 (0.85, 1.79)	0.275	0.9 (0.50, 1.44)	0.544
Prenatal care initiation (month)				
1-3	Reference	Reference	Reference	Reference
4-6	1.6 (1.36, 1.95)	0.000	1.3 (1.02, 1.65)	0.034
7-9	2.4 (1.87, 3.20)	0.000	1.2 (0.76, 1.92)	0.426
None	2.3 (1.20, 4.51)	0.012	1.7 (0.64, 4.60)	0.287

Associations between maternal education and other characteristics with the occurrence of maternal ICU admissions

Table 4 shows the adjusted and unadjusted associations between maternal ICU admissions and selected characteristics. We did not find a statistically significant relationship between maternal educational attainment and maternal ICU admission or any in the unadjusted and adjusted models. Results from the adjusted analyses showed that mothers aged 20-24 have lower odds of ICU admission when compared to mothers aged 25-29 (OR = 0.73, 95% CI = 0.53, 1.00, p = 0.048). Mothers with a BMI greater than 30.0 were found to have higher adjusted odds of maternal ICU admission than their counterparts with BMIs of 18.5-24.9 (OR = 1.31, 95% CI = 1.02, 1.68, p = 0.034). Women with partners with a bachelor's degree or less (OR = 0.79, 95% CI = 0.63, 0.98) and a master's degree or more (OR = 0.52, 95% CI = 0.33, 0.80) have lower adjusted odds of ICU admission. Starting prenatal care in the second trimester as opposed to the first trimester was also correlated with higher adjusted odds of maternal ICU admission (OR = 1.30, 95% CI = 1.01, 1.65).

**Table 4:** Unadjusted and adjusted associations between maternal education, selected characteristics, and maternal ICU admission Unadjusted and adjusted analyses of maternal ICU; binary logistic regression for calculation of unadjusted and adjusted odds ratios (ORs) and corresponding 95% confidence intervals (CIs) BMI: body mass index; WIC: Women, Infants, and Children Program

Characteristics	Unadjusted		Adjusted	
	OR (95% CI)	p-value	OR (95% CI)	p-value
Maternal education				
High school	Reference	Reference	Reference	Reference
Some college	0.8 (0.68, 0.94)	0.007	0.9 (0.76, 1.19)	0.667
Master's	0.8 (0.62, 1.07)	0.145	1.2 (0.85, 1.78)	0.271
Maternal age				
18-19	1.5 (0.90, 2.48)	0.125	1.6 (0.86, 2.83)	0.146
20-24	0.9 (0.75, 1.23)	0.748	0.7 (0.53, 1.00)	0.048
25-29	Reference	Reference	Reference	Reference
30-34	1.1 (0.96, 1.35)	0.178	1.1 (0.89, 1.32)	0.427
Maternal race				
Non-Hispanic White	Reference	Reference	Reference	Reference
Non-Hispanic Black	1.4 (1.08, 1.71)	0.009	1.2 (0.87, 1.61)	0.291
Hispanic	1.1 (0.87, 1.27)	0.609	1.0 (0.81, 1.34)	0.735
Other	1.1 (0.85, 1.35)	0.552	1.3 (0.99, 1.78)	0.057
Maternal nationality				
US-born	Reference	Reference	Reference	Reference
Non-US-born	0.9 (0.78, 1.11)	0.434	0.9 (0.74, 1.21)	0.676
Maternal BMI				
Underweight	1.4 (0.77, 2.65)	0.257	1.4 (0.66, 2.83)	0.389
Normal	Reference	Reference	Reference	Reference
Overweight	1.3 (1.04, 1.67)	0.021	1.3 (0.99, 1.69)	0.058
Obese	1.4 (1.10, 1.68)	0.004	1.3 (1.02, 1.68)	0.034
Father's education				
High school	Reference	Reference	Reference	Reference
Some college	0.8 (0.67, 0.96)	0.016	0.8 (0.63, 0.98)	0.034
Master's	0.6 (0.39, 0.82)	0.003	0.5 (0.33, 0.80)	0.003
Maternal WIC status				
Yes	1.2 (0.98, 1.35)	0.080	1.1 (0.86, 1.34)	0.539
No	Reference	Reference	Reference	Reference
Maternal pay (insurance)				
Medicaid	1.21 (1.03, 1.42)	0.021	0.92 (0.72, 1.16)	0.477
Private insurance	Reference	Reference	Reference	Reference
Self-pay	1.61 (1.07, 2.42)	0.023	1.28 (0.77, 2.12)	0.338
Other	1.06 (0.69, 1.63)	0.795	0.85 (0.50, 1.44)	0.544
Prenatal care initiation (month)				
1-3	Reference	Reference	Reference	Reference
4-6	1.4 (1.10, 1.65)	0.004	1.3 (1.01, 1.65)	0.034
7-9	1.2 (0.79, 1.73)	0.445	1.2 (0.76, 1.92)	0.426
None	4.5 (2.73, 7.44)	0.000	1.7 (0.64, 4.61)	1.070

## Discussion

This study explored the potential relationship between lower maternal education attainment and the risk of adverse health outcomes in both mother and child in pregnant women with gestational diabetes in the United States. Outcomes assessed included maternal ICU admissions and congenital abnormalities, namely, anencephaly, meningomyelocele/spina bifida, cyanotic congenital heart disease, congenital diaphragmatic hernia, omphalocele, gastroschisis, limb reduction defect, and cleft lip with or without cleft palate. Our data revealed that maternal education levels were not associated with birthing a child with at least one congenital anomaly or with maternal ICU admission.

Our findings contrast with the current scientific literature regarding maternal education and the risk of negative health outcomes in children of pregnant women with gestational diabetes. For instance, a cohort study of 428,715 live births conducted in Italy from 2005 to 2010 reported that mothers with an intermediate level of education were associated with lower odds of severe congenital abnormalities (OR = 0.94, 95% CI = 0.91, 0.98) compared to mothers with lower levels of education. However, a higher education level was not associated with the odds of severe congenital abnormalities (OR = 1.01, 95% CI = 0.97, 1.06) [11]. Yet, while this study had a large sample, it was not restricted to women experiencing gestational diabetes. Additionally, the outcome assessed included severe malformations only as defined by the EUROCAT classification. Our study defined congenital anomalies as anencephaly, meningomyelocele/spina bifida, cyanotic congenital heart disease, congenital diaphragmatic hernia, omphalocele, gastroschisis, limb reduction defect, and cleft lip with or without cleft palate. Finally, the present study considered a more comprehensive set of variables such as maternal BMI, socioeconomic status, and paternal education. All these differences could have contributed to the inconsistencies in the findings of the present study.

Several other studies have further emphasized the relevance of maternal education in the context of gestational diabetes mellitus. Most recently, a large population-based cohort study using US vital statistics data from 2016 to 2019 evaluated over 850,000 women with gestational or pregestational diabetes to assess the impact of maternal education on perinatal outcomes. Compared to individuals with a college education or higher, those with some college (aRR = 1.08, 95% CI = 1.07, 1.09), HS (aRR = 1.06, 95% CI = 1.04, 1.07), or less than HS education (aRR = 1.05, 95% CI = 1.03, 1.07) had modestly increased risks of adverse neonatal outcomes, including large for gestational age, low Apgar scores, and prolonged assisted ventilation, as well as elevated maternal risks such as ICU admission, transfusion, and unplanned hysterectomy [12]. Despite similar outcomes being examined in both studies, our analysis did not observe a statistically significant association between maternal education and ICU admission or congenital anomalies, which may reflect differences in sample size and population characteristics due to the study spanning a larger time frame from 2016 to 2019.

Similarly, a recent case-control study by Topkara and Soysal included 119 pregnant women diagnosed with gestational diabetes who gave birth between August 2022 and January 2024 [13]. The study assessed the impact of participation in a diabetes education program on maternal and neonatal outcomes. Participants were divided into two groups: 57 received diabetes education and 62 did not. The study found that women who did not receive diabetes education had significantly higher hemoglobin A1c levels (p = 0.013) and greater total gestational weight gain (p = 0.015) [13]. Additionally, their infants had higher birth weights (p = 0.005) and a higher incidence of macrosomia, particularly among those requiring insulin therapy [13]. In parallel, education level may also influence long-term maternal health. A retrospective cohort study of women with gestational diabetes followed across 25 Portuguese health institutions between 2008 and 2012 found that those with only primary education had more than twice the odds of developing persistent postpartum glucose metabolism disorders compared to women with higher education (OR = 2.37, 95% CI = 1.69, 3.32) [14]. This highlights the broader implications of education on health and health literacy in the gestational diabetes population.

Together, the aforementioned studies demonstrate the impact of formal schooling, and targeted diabetes education may play a protective role in maternal and neonatal outcomes. Moreover, a population-based cohort conducted in Rotterdam, the Netherlands, including mothers with delivery dates between April 2002 and January 2006, identified a strong association between lower educational attainment and increased odds of developing gestational diabetes compared to those with higher education (OR = 3.07, 95% CI = 1.37, 6.89), independent of ethnicity, maternal age, and lifestyle factors [15]. Although our study focused on outcomes following gestational diabetes diagnosis, the conclusions drawn from this study reinforce the role of maternal education as a potential social determinant of health and an important piece in the complex puzzle of risk factors contributing to the development of gestational diabetes.

Interestingly, paternal education was associated with the outcomes assessed. Fathers with higher levels of education, such as a master's degree, have a slightly lower proportion of newborns with congenital anomalies compared to fathers with HS or some college education. After adjusting for other factors, these associations remain significant for fathers with "some college" and "master's," indicating that higher education of the father is linked with reduced odds of congenital anomalies. Paternal education was also associated with maternal ICU admission in a similar pattern.

At least two studies reported an association between paternal education levels and perinatal complications. A study utilizing data from the 2006 Canadian Birth-Census Cohort, focusing on singleton births, noted that lower paternal education was associated with increased risks of adverse birth outcomes, including preterm birth, small-for-gestational-age (SGA) birth, stillbirth, and infant mortality, independent of maternal education, age, marital status, parity, ethnicity, and nativity [16]. The second study conducted by Olesen was a Danish population-based cohort study that aimed to investigate the association between maternal and paternal educational levels, household income, and the risk of giving birth to a baby with a congenital anomaly in 19,874 primiparous singleton women between 1991 and 1998. About 5.2% of babies reported congenital anomalies. Women with less than 10 years of schooling had nearly a threefold increased risk compared to those with more than four years of higher education (OR = 2.9, 95% CI = 1.8, 4.6). Higher paternal educational level and household income were also associated with congenital anomalies in offspring, although to a lesser extent [17]. Whether the higher frequency of congenital anomalies or adjustments for income explain differences in results is yet to be tested. Regarding our findings of higher paternal education reducing the odds of maternal ICU admission, there is limited literature reporting on the impact of paternal education on maternal outcomes during pregnancy that we could use for comparison.

It is worth noting that fathers with higher education also tend to have partners with higher education levels. For instance, 60% of mothers with at least some college education have husbands with similar education levels. Similarly, mothers with a master's degree have husbands with at least some college education (51.4%) or a master's degree or higher (40.4%). Thus, a certain level of correlation might have explained the lack of independent associations for mothers' education once both variables were entered into the model. To further explore whether such findings indicate collinearity, analyses comparing models omitting the variable for the father's education did not significantly affect our results (maternal education was not associated with the odds of congenital anomalies or maternal ICU hospitalization, data not shown). We also found that about 11% of the mother-child dyads analyzed had missing data on paternal education. We consider the possibility that missing data could indicate, at some frequency, the absence of a paternal figure in the family. Analyses conducted, including those missing paternal education data as a subcategory (as opposed to excluding them from analyses), did not affect the results for the association of maternal or paternal education and the outcomes assessed. Further research is needed to investigate the relationship between father and mother education interaction, how they potentially relate to the sources of family income, and their impact on congenital anomalies in children of mothers with gestational diabetes. The potential collinearity can be further explored with advanced modeling techniques such as variance inflation factor (VIF) analysis and correlation matrices.

The present study has some limitations that need to be addressed. For instance, relying on data from the NVSS limited the availability and details on potential variables. The dataset did not have information on key factors, such as family income and the role of the mother/father as the main provider. Thus, residual confounding related to socioeconomic status or access to healthcare is possible. Also, it is likely that the occurrence of congenital malformations was underestimated, as the records were only for gestations for which there was a live birth. Abortions and stillbirths resulting from congenital anomalies were missed. On the other hand, relying on data from the NVSS/National Center for Health Statistics standardized reporting approach provided precise and dependable real-time data. Thus, the data collected is the most accurate and generalizable for the US population's births. Of note, updating findings using more recent data as it becomes available would enhance generalizability and allow for the evaluation of temporal trends.

Another limitation relates to the categorization of maternal education. Due to constraints in the dataset and to maintain adequate sample sizes within subgroups, we categorized maternal education into three broad levels. In doing so, we combined individuals with some college education and those with a bachelor’s degree into a single group. This categorization, while methodologically practical, may limit the interpretability of our findings by potentially masking meaningful differences in health outcomes between these distinct educational subgroups. Future studies with more granular education data may be better positioned to explore these nuances.

## Conclusions

Gestational diabetes is a common complication in pregnant women that can lead to serious health risks for both the mother and fetus during pregnancy and labor. Our study aimed to contribute to the existing body of literature and advocate for improved education for mothers diagnosed with gestational diabetes, ultimately enhancing maternal and newborn outcomes. Additionally, we sought to provide health professionals with valuable insights for tailoring treatment plans for high-risk populations, such as those with gestational diabetes. While maternal education was not independently associated with the selected outcomes assessed, our findings suggest that social determinants of health, including paternal barriers to health literacy, may influence patient outcomes. These results underscore the need for a more comprehensive approach to patient education and healthcare interventions that address broader socioeconomic factors affecting maternal and fetal health.
